# Iron in breastfed infants and behavior at 3 years: a randomized trial

**DOI:** 10.1038/s41598-026-56401-z

**Published:** 2026-06-16

**Authors:** Ludwig K. Svensson, Grzegorz Chmielewski, Magnus Domellöf, Zofia Konarska, Małgorzata Pieścik-Lech, Cornelia Späth, Hania Szajewska, Anna Chmielewska

**Affiliations:** 1 Department of Clinical Sciences, Pediatrics, Umeå University, Umeå, Sweden; 2 Department of Clinical Sciences, Obstetrics and Gynecology, Umeå University, Umeå, Sweden; 3 Department of Pediatrics, Medical University of Warsaw, Warsaw, Poland

**Keywords:** Diseases, Health care, Medical research

## Abstract

Breastfed infants are particularly vulnerable to iron deficiency, which is associated with impaired neurodevelopment. Iron supplementation reduces behavioral problems in certain risk groups, but effects in healthy infants are uncertain. This randomized, double-blind, placebo-controlled trial evaluated whether daily low-dose iron supplementation (7, 10, or 15 mg micronized microencapsulated ferric pyrophosphate, approximating 1 mg/kg, from 4 to 9 months of age) affects behavioral outcomes at 3 years. The study was conducted in Warsaw, Poland and Umeå, Sweden and included full-term, healthy, predominantly breastfed infants who were non-anemic at baseline. Behavioral problems were assessed using the Child Behavior Checklist (CBCL) at 3 years as a pre-specified secondary outcome. Of the 221 participants randomized, 133 (60%) completed the CBCL. Iron supplementation compared to placebo significantly reduced CBCL externalizing T-scores (mean [SD], 45.6 [8.5] vs. 48.6 [8.9], adjusted *P* = .006). Lower scores were also observed for aggressive behaviors (adjusted *P* = .0329). This pre-specified secondary outcome analysis found that low-dose iron supplementation in predominantly breastfed healthy infants between 4 and 9 months of age may contribute to fewer behavioral problems at 3 years of age. These findings suggest a potential beneficial effect, though cautious interpretation is warranted until replicated in future studies.

**Trial registration**: clinicaltrials.gov; identifying number: NCT02242188; URL: http://www.clinicaltrials.gov/study/NCT02242188; Date of registration: 2014-09-14.

**Supplementary Information:**

The online version contains supplementary material available at 10.1038/s41598-026-56401-z.

## Introduction

Iron is essential for normal brain development and plays a critical role in neurometabolism, myelination, and neurotransmitter synthesis^1–5^. Early iron deficiency (ID) has been associated with long-lasting behavioral problems^6–8^. Iron supply to the brain may decrease before ID is detectable using traditional lab analyses^9,10^.

Young children are particularly vulnerable to ID^11,12^. Although prevalence is highest in low-income countries, approximately 20% of children under 3 years in Europe and North America are affected^11,13,14^. At the same time, 23% of American preschoolers experience emotional-behavioral disorders, including internalizing behaviors (1 in 10), and externalizing behaviors such as Attention Deficit and Hyperactivity Disorder (ADHD, 1 in 5)^15^. ADHD and Autism Spectrum Disorder (ASD) are more frequent in populations at risk of ID (e.g. children born preterm)^16–18^, suggesting ID may contribute to neurodevelopmental vulnerability.

The occurrence and severity of ADHD has been associated with ID in 22 of 30 studies and 4 of 4 systematic reviews identified by a recent scoping review^19^. Two identified small randomized controlled trials (64 participants in total, reported that iron supplementation was beneficial in reducing ADHD symptoms. Given the high prevalence of neurodevelopmental conditions and the potential causative role of ID, preventing ID is of high priority.

Current guidelines recommend delayed cord clamping and timely introduction of iron-rich complementary foods to reduce the risk of ID^13^. Prophylactic iron supplementation is recommended in well-defined risk groups such as infants born preterm or/and with low birth weight^13,14^. Whether healthy infants, including those exclusively breastfed, should be considered a risk group for ID, and thus universally supplemented, has been controversial^20–22^. Exclusive breastfeeding is recommended for the first 6 months of life^23^. However, prolonged breastfeeding without sufficient iron intake from complementary foods will lead to ID^20,22,24,25^. Evidence regarding developmental benefits in healthy, breastfed infants is scarce and recommendations are diverging^26,27^. While the American Academy of Pediatrics advises iron supplementation (1 mg/kg/day) from four months for predominantly breastfed infants^14^, the European Society for Pediatric Gastroenterology, Hepatology and Nutrition does not endorse universal supplementation in populations with a low prevalence of iron deficiency anemia (IDA)^13^.

The Supplementing Iron and Development in Breastfed Infants (SIDBI) study is a double-blind, placebo-controlled RCT designed to examine the effects of early iron supplementation in predominantly or exclusively breastfed infants. This pre-specified secondary data analysis aims to evaluate the effects of iron supplementation on behavioral outcomes. The primary outcome, psychomotor development at 12 months, showed no benefit from iron supplementation^28^.

## Methods

### Study design and participants

SIDBI was a pragmatic, double-blind, parallel-group RCT conducted between December 2015 and May 2020 at Umeå University Hospital, Sweden, and the Medical University of Warsaw, Poland. Follow up was completed in May 2023. The primary outcome, effect of iron supplementation on psychomotor development at 12 months of age, and adverse events have previously been published^28^.

The trial protocol was published before the trial’s initiation^29^. Ethical approval was granted by the Bioethical Committee of the Medical University of Warsaw (174/2014) and the Umeå University Ethical Review Board (2018/281 − 31). We adhered to the Consolidated Standards of Reporting Trials guidelines^30^.

Eligible participants were healthy, singleton infants born at term (37–42 weeks gestation) with birth weight > 2500 g, who were exclusively or predominantly breastfed (> 50% of daily intake) and non-anemic (hemoglobin > 10.5 g/dL) at 4 months. Exclusion criteria were major illnesses, food allergies, and congenital anomalies. Written informed consent was obtained from caregivers before randomization, and the study was conducted in accordance with all relevant guidelines and regulations.

### Randomization and blinding

Participants were assigned to the intervention (iron supplementation, Group A) or placebo (Group B) using a computer-generated randomization list stratified by sex, with assignments in blocks of 10. The randomization key was stored in a sealed envelope within a safe located in the administration office at the Medical University of Warsaw. Study products arrived in unmarked boxes labeled “A” or “B” and were relabeled with participants’ study IDs by a non-affiliated individual. Blinding of researchers, psychologists, research nurses, and caregivers was maintained until all assessments were completed.

### Intervention

Participants consumed one sachet of 1 mg/kg of iron (micronized microencapsulated ferric pyrophosphate) or placebo (maltodextrin) daily between 4 and 9 months of age. Caregivers were instructed to mix the sachets with 10 mL lukewarm water or breast milk. Three iron doses were used: 7 mg, 10 mg, and 15 mg for infants weighing < 7 kg, 7–10 kg, and > 10 kg, respectively. Iron and placebo sachets were identical in appearance, taste, and texture. Caregivers returned unused sachets, and participants were considered compliant if < 25% were returned.

### Outcome measures

Behavior at 3 years of age was assessed using the Achenbach’s Child Behavior Checklist (CBCL) for ages 1.5–5 years. It was a secondary outcome in the SIDBI trial^29^.

### Sample size calculation

The sample size of the SIDBI trial was calculated to detect a 5-point difference in the Bayley II-equivalent of Bayley III motor composite scores at 12 months of age (main outcome). With a power of 80% and an α of 0.05, 220 participants were required, assuming a 12-point SD and 20% dropout.

#### Data collection

Details on data collection were published elsewhere^29^. The CBCL is a validated psychometric instrument for assessing behavioral problems in young children^31^. Caregivers independently completed the CBCL and returned it to study staff during the child’s 3-year visit. It contains 100 questions rated on a 3-point scale: 0 for “not true”, 1 for “sometimes true”, and 2 for “very/often true”. These questions form three broadband scales: internalizing, externalizing, and total problem scores. The internalizing broadband scale comprises four subscales: emotionally reactive, anxious/depressed, somatic complaints, and withdrawn behavior. The externalizing broadband scale includes two subscales: attention problems and aggressive behavior. Adding these two broadband scales with the subscales for sleep problems and other problems produces the total score.

T-score cutoffs from a US pediatric population define clinically and subclinically significant problems as T > 63 and T > 59, respectively^31^. Due to lower CBCL scores in Swedish children, a Swedish cohort was used to apply an additional > 90th percentile cutoff and standardize subscale scores^32^. No published Polish normative data is available for the CBCL version used. Validated translations of the CBCL were used in both countries.

Venous blood samples were collected from children at 4 and 12 months of age. Samples treated with EDTA were analyzed for complete blood cell count in the hospital laboratory. Serum samples were frozen at −80 °C for later analyses of high-sensitive C-reactive protein (CRP) through the human CRP Quantikine ELISA (R&D Systems) and ferritin concentrations using the Human Ferritin ELISA Kit (Thermo Fisher Scientific). All serum samples were analyzed at Umeå University Hospital, Sweden. Children with anemia (hemoglobin < 10.5 g/dL) were referred to a pediatrician for evaluation.

### Study oversight

Recruitment began in 2015 in one delivery ward and several well-baby clinics in Warsaw and paused at reaching 50% of the target sample size in November 2018 due to slow progress. The remaining participants were recruited in Umeå, Sweden between 2018 and 2020. The COVID-19 pandemic reduced follow-up rates in Poland but had minimal impact in Sweden.

### Statistical analysis

All analyses were conducted based on the intention-to-treat (ITT) principle. While the formal definition of ITT requires the inclusion of all randomized participants^33^, 39.8% of participants were excluded due to missing data, preventing full adherence. However, the analysis retained the core principles of ITT by analyzing participants according to their randomization group, ensuring methodological consistency. The term ITT was used to describe this methodology and ensure a broader understanding of the applied analyses. For exploratory analyses of associations between iron status and behavioral scores, the intervention groups were combined into one cohort.

Statistical analyses were performed in R software (version 4.1.2) by an external statistician. Missing data was assumed to follow a combination of missing completely at random and missing at random and addressed using multiple imputation (m = 5) with predictive mean matching (mice R package). The imputation model included CBCL variables, nationality, sex, and birth weight. Statistical outcomes were pooled using mice after and psfmi R packages. To account for possible missing not at random scenarios, sensitivity tipping point analyses were performed for CBCL broad scales. Missing outcomes in the target group (iron and placebo separately) were systematically shifted across SD units, while outcomes in the other group were held at their observed mean.

Normality was verified with Shapiro-Wilk test, skewness, and kurtosis. Variance homogeneity was assessed with Levene’s test. Comparisons between intervention groups were performed with Student’s t-test, Mann-Whitney U test, Pearson’s chi-square test or Fisher’s exact test as appropriate. Linear regression models comparing CBCL outcomes were adjusted for sex, nationality, birth weight and breastfeeding. P-values for syndrome scales and the broadband scales were corrected using the Benjamini-Hochberg procedure to adjust for multiple comparisons. Baseline characteristics were compared in SPSS v29.0.2.0 (IBM) using t-tests after verifying normality. Outcomes were considered significant if *P* <.05.

## Results

**Fig. 1:**
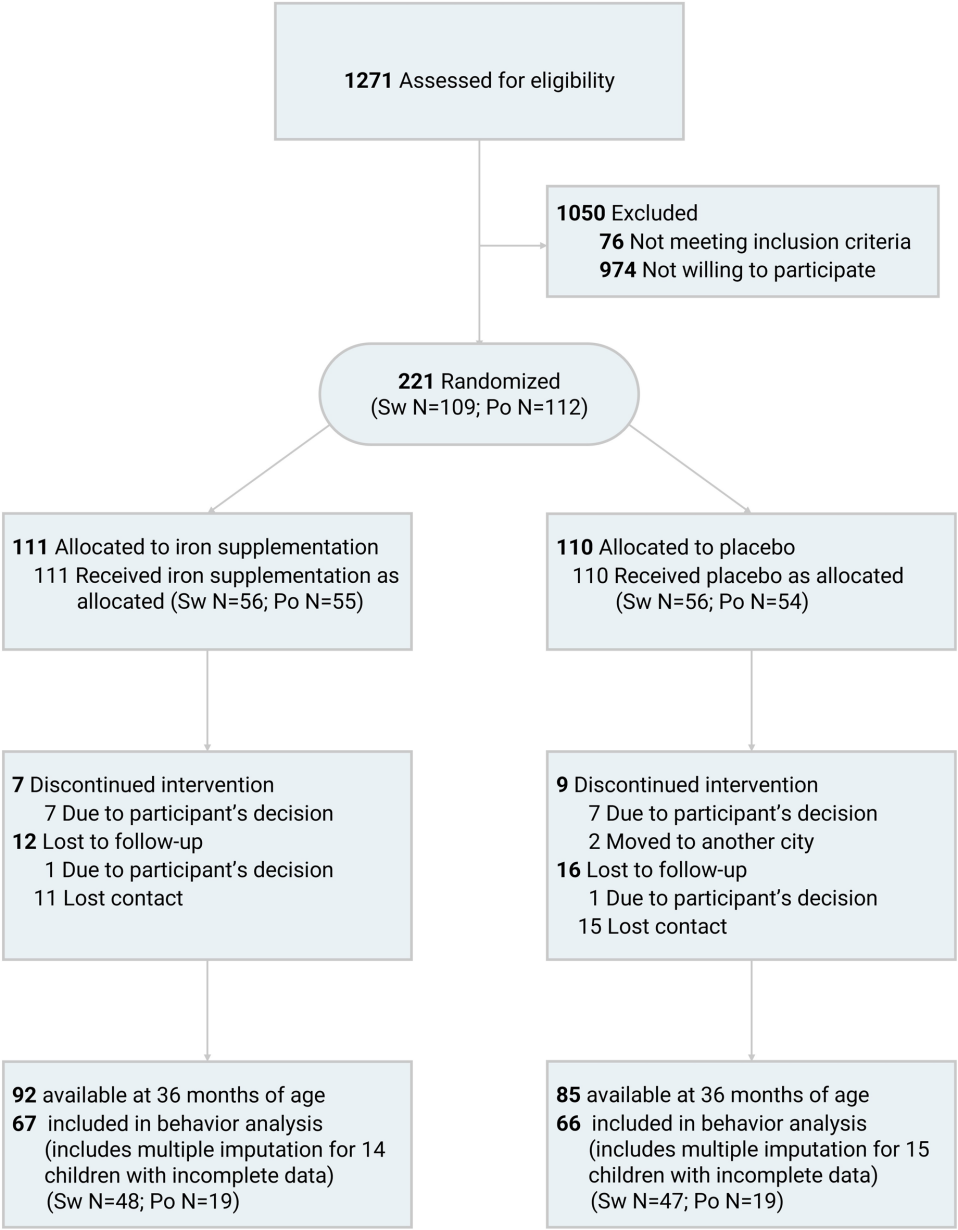
Trial flowchart. Flowchart of the 221 trial participants. At 3 years, 177 (80%) remained (iron: *n* = 92, placebo: *n* = 85). Data from the Child Behavior Checklist were available for 133 (60%). Compliance with the intervention was 84%. Both ITT (*n* = 133) and PP (*n* = 109) analyses were conducted.

Of the 221 participants randomized at 4 months, 92/111 (83%) in the iron group and 85/110 (77%) in the placebo group remained in the study at 36 months. Of these, 133 children (60%) had available CBCL data (Fig. 1). Background and baseline characteristics by randomization group are presented in Table 1. Based on available data, the compliance rate to the intervention was 84% overall (*n* = 221, 22% missing data on compliance), 88% in the iron supplementation group (23% missing) and 81% in the placebo group (20% missing).

**Table 1:** Background and baseline characteristics of the total study population and participants with behavior data, presented by intervention group.

Characteristic, *n* (%)	Whole population^a^		With available^b^ CBCL data^c^	
	Iron (*n* = 111)	Placebo (*n* = 110)	Iron (*n* = 67)	Placebo (*n* = 66)
Nationality				
Polish	56 (50.5)	56 (50.9)	19 (28.4)	19 (28.8)
Swedish	55 (49.5)	54 (49.1)	48 (71.6)	47 (71.2)
Sex				
Male	55 (49.5)	55 (50.0)	31 (46.3)	29 (43.9)
Female	56 (50.5)	55 (50.0)	36 (53.7)	37 (56.1)
Infant characteristics, mean (SD)				
Gestational age	39.5 (1.2)	39.4 (1.4)	39.7 (1.1)	39.7 (1.3)
Birth weight	3549.5 (408.7)	3553.1 (389.1)	3553.4 (403.0)	3668.3 (381.5)
Birth head circumference	35 (1.3)	35 (1.4)	35 (1.4)	35 (1.2)
Maternal education level				
Compulsory school	9 (9.1)	13 (13.4)	7 (10.8)	11 (17.2)
Upper secondary or college	8 (8.1)	6 (6.2)	8 (12.3)	4 (6.3)
University or higher	82 (82.8)	78 (80.4)	50 (76.9)	49 (76.6)
Paternal education level				
Compulsory school	3 (3.0)	2 (2.1)	2 (3.1)	1 (1.6)
Upper secondary or college	27 (27.3)	29 (30.2)	21 (32.3)	22 (34.9)
University or higher	69 (69.7)	65 (67.7)	42 (64.6)	40 (63.5)
Breastfeeding				
Breastfeeding at 6 months	74 (78.7)	64 (68.8)	54 (85.7)	51 (79.7)
Exclusively breastfed at 6 months	57 (60.6)	46 (49.5)	39 (61.9)	34 (53.1)
Breastfeeding at 9 months	42 (53.2)	38 (47.5)	36 (66.7)	31 (50.8)
> 50% breastfed at 9 months	12 (28.6)	11 (28.9)	10 (27.8)	9 (29.0)
Iron deficiency^d^ at 12 months	5 (6.4)	9 (14.3)	0 (0)	3 (7.5)
Iron deficiency anemia^e^ at 12 months	1 (1.5)	1 (1.9)	0 (0)	0 (0)

^a^Available data on each variable in iron/placebo group: 110/111 (nationality, sex), 110/109 (birth weight), 108/109 (gestational age), 99/97 (maternal education, birth head circumference), 99/96 (paternal education), 94/93 (breastfeeding at 6 months), 86/77 (ferritin), 82/73 (C-reactive protein), 80/70 (hemoglobin), 79/80 (breastfeeding at 9 months), 78/63 (ID at 12 months), 68/53 (IDA at 12 months).

^b^Complete CBCL was available for 104 participants (53/51); multiple imputation was used for those with incomplete CBCL data (14/15).

^c^Available data on each variable in iron/placebo group: 67/66 (gestational age), 65/64 (maternal education), 65/63 (paternal education), 63/66 (birth weight), 63/64 (breastfeeding at 6 months), 63/56 (birth head circumference), 54/61 (breastfeeding at 9 months), 54/50 (ferritin), 52/48 (C-reactive protein), 51/40 (ID at 12 months), 49/47 (hemoglobin), 44/36 (IDA at 12 months).

^d^Defined as ferritin concentration < 12 ng/mL; samples with C-reactive protein > 0.5 mg/dL are excluded (*n* = 13); percentages are calculated based on available data.

^e^Defined as a combination of iron deficiency in combination with hemoglobin < 10.5 g/dL; percentages are calculated based on available data.

Of the 133 participants with available CBCL data, 29 (22%) provided incomplete CBCL questionnaires. Internal consistencies for each CBCL scale are presented in eTable 1, Supplement. Twelve participants received a CBCL questionnaire missing the second page, covering questions 55–100. Overall, missing CBCL data ranged between 0% and 13% among participants with CBCL outcomes present. Following multiple imputation for incomplete CBCL data (*n* = 29), analyses based on ITT (*n* = 133) and per-protocol (PP) (*n* = 109) were conducted. When comparing baseline infant characteristics, participants with available CBCL data had significantly higher mean birth weight and gestational age compared to the 88 participants with missing CBCL data (mean [SD]; 3610 [395] grams vs. 3460 [387] grams, *P* =.01, and 40 [1] weeks vs. 39 [1] weeks, *P* <.001, respectively).

The overall mean age for completing the CBCL form was 3.02 (SD 0.18) years (*n* = 107), 3.02 (SD 0.17) years for the intervention group and 3.01 (SD 0.18) years for the placebo group.

In the ITT analysis of broadband scales (Table 2), mean CBCL total T-score and externalizing T-score were significantly lower in the iron group compared to placebo (mean [SD]; 45.0 [8.0] vs. 47.7 [8.2], *P* =.01, and 45.6 [8.5] vs. (48.6 [8.9]), P *<*.001, respectively), both in unadjusted analysis and after adjusting for sex, nationality, birth weight and exclusive breastfeeding at 6 months. Differences between groups in internalizing T-scores, and the proportions above the clinical and subclinical cutoffs were not significant. Results were similar in PP analysis (eTable 2, Supplement). After correcting for multiple comparisons, the difference in externalizing T-scores was still significant in ITT analysis, while both total T-score and externalizing T-score differences remained significant in PP analysis.

**Table 2:** CBCL broadband scores and cutoffs^a^ between study groups, intention-to-treat analysis.

CBCL scale^b^	Iron (*n* = 67)	Placebo (*n* = 66)	MD or RR (95% CI)^c^	*P* ^d^	*P* ^e^	*P* ^f^
Total T-score	45.0 (8.0)	47.7 (8.2)	−2.75 (−5.52 to 0.02)	**0.01** ^1^	0.08	0.05
Total T-score above clinical cutoff	0 (0)	2 (2.6)	NA	.24^2^	0.47	
Total T-score above subclinical cutoff	2 (3.2)	5 (8.2)	0.40 (0.08 to 2.08)	.27^2^	0.47	
Total T-score above Swedish cutoff	4 (6.2)	9 (13.3)	0.47 (0.15 to 1.48)	.23^3^	0.47	
Internalizing T-score	43.9 (9.3)	46.9 (8.7)	−3.00 (−6.13 to 0.13)	.12^1^	0.32	0.07
Internalizing T-score above clinical cutoff	0 (0.0)	1 (1.5)	NA	.5^2^	0.61	
Internalizing T-score above subclinical cutoff	4 (6.1)	8 (11.7)	0.52 (0.15 to 1.81)	.51^3^	0.61	
Internalizing T-score above Swedish cutoff	5 (8.2)	10 (14.5)	0.57 (0.18 to 1.80)	.43^3^	0.58	
Externalizing T-score	45.6 (8.5)	48.6 (8.9)	−2.93 (−5.89 to 0.03)	**< 0.001** ^**1**^	**0.006**	**0.04**
Externalizing T-score above clinical cutoff	2 (3.0)	5 (7.6)	0.39 (0.08 to 1.96)	.27^2^	0.47	
Externalizing T-score above subclinical cutoff	4 (6.5)	5 (7.6)	0.86 (0.24 to 3.10)	.74^2^	0.78	
Externalizing T-score above Swedish cutoff	4 (6.5)	5 (7.6)	0.86 (0.24 to 3.10)	.74^2^	0.78	

MD – Mean difference (iron vs. placebo groups); RR – relative risk (iron vs. placebo groups); CI – confidence interval; NA – not applicable.

^a^Clinical cutoff: Broadband scale cutoffs: clinical (T-Score > 63), subclinical (T-Score > 59); Swedish cutoff: >90th percentile of a Swedish reference population (30).

^b^Numeric variables are presented as mean (SD); categorical variables are presented as pooled % of group based on pooled % and total number of patients in iron/placebo group.

^c^MD for numeric values, RR of scoring above cutoff for categorical variables. Note: Discrepancies between P values and CIs, with some CIs crossing zero despite statistical significance, may occur due to multiple imputation.

^d^Groups were compared using (1) Student’s t-test; (2) Fisher’s exact test; (3) Pearson’s chi-square test, as appropriate.

^e^P values adjusted using the Benjamini-Hochberg method.

^f^Adjusted for sex, nationality, birth weight, and exclusive breastfeeding at 6 months. Groups were compared using linear regression models with the CBCL broadband score as the dependent variable, intervention group as the independent variable, and sex, nationality, birth weight, and exclusive breastfeeding at 6 months as covariates. Estimates were pooled across multiply imputed datasets.

**Fig. 2:**
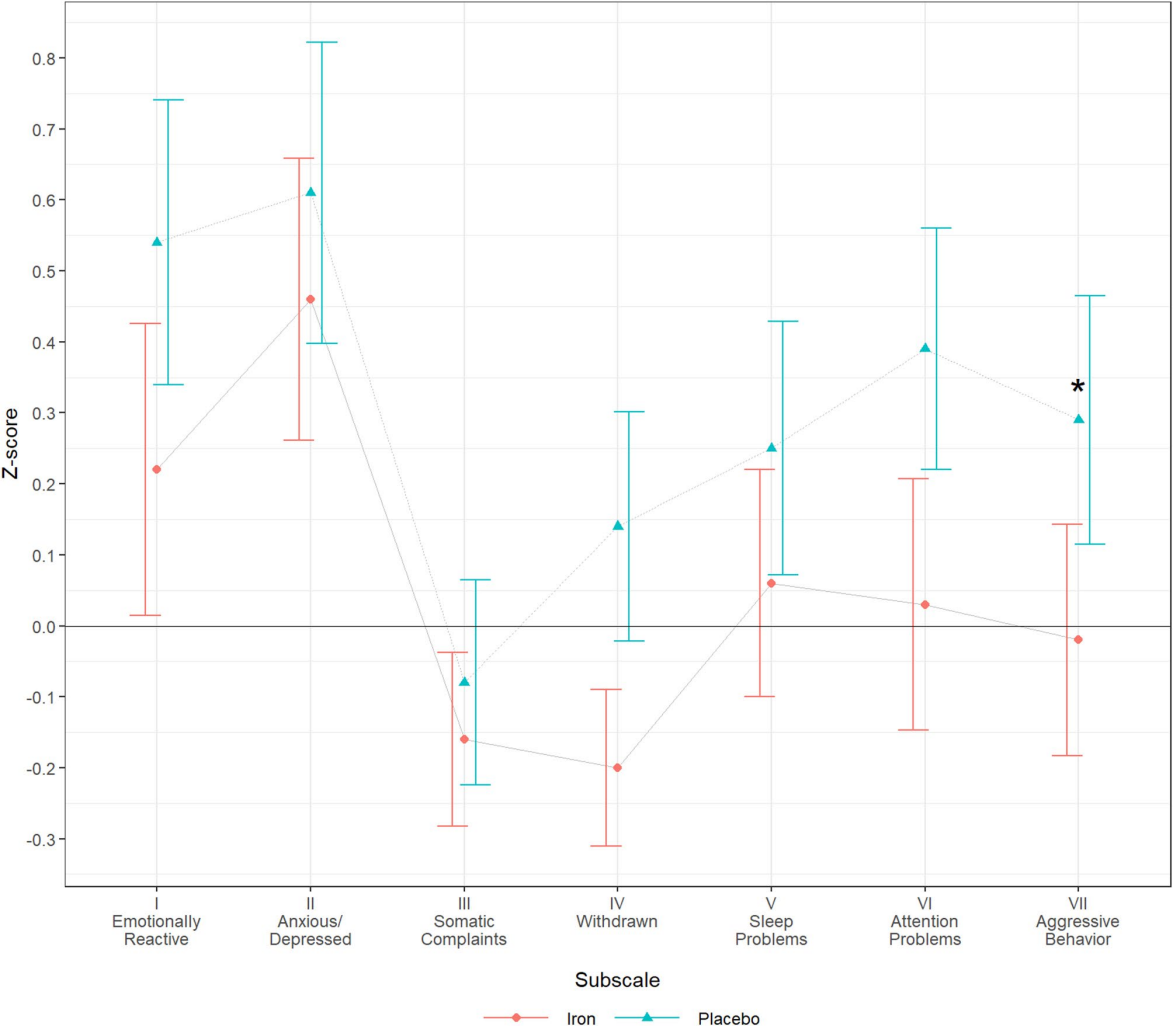
Child Behavior Checklist subscale profile between randomization groups in intention-to-treat analysis. Child Behavior Checklist subscale scores at 3 years of age in children randomized to receive iron (1 mg/kg/day) or placebo from 4 to 9 months of age. Z-scores were calculated using pooled means of Child Behavior Checklist subscale scores and standardized to a Swedish reference population^32^. Higher scores indicate more behavioral problems. * denotes significant differences (*P <*.05) after adjustment using the Benjamini-Hochberg method. Note: 95% confidence intervals are based on pooled estimates from multiple imputation and may overlap despite statistically significant mean differences.

In the ITT analysis of subscales, participants in the iron group had significantly lower withdrawn scale scores (median [IQR]; −0.6 [−0.6 to 0.1] vs. 0.1 [−0.6 to 0.1], *P* =.02), attention problems scores (−0.3 [−1.0 to 0.7] vs. 0.3 [−0.3 to 1.0], *P* =.02), and aggressive behavior scores (mean [SD]; 0.0 [1.0] vs. 0.3 [1.0], *P* =.003) compared to the placebo group. After adjustment for multiple comparisons, the difference in raw aggressive behavior scores remained statistically significant. Pooled results are presented in Fig. 2, and raw results in eTable 3, Supplement. In the PP analysis, mean aggressive behavior was significantly lower in the iron supplemented group compared to the placebo even after correcting for multiple testing. (eTable 2, Supplement).

To examine the effect of intervention assuming that those lost to follow up were missing not at random, we performed sensitivity analysis testing extreme scenarios. For total and externalizing T-scores, the original significant differences (behavioral scores lower in iron compared to placebo) were generally robust: significance was maintained for moderate shifts in either group (up to ~ 0.3–0.4 SD for iron drop-outs, above ~ −0.3 SD for placebo drop-outs). Larger shifts led to loss of significance or, under highly extreme and clinically unlikely scenarios, reversal of the effect of direction. For internalizing T-scores, the original non-significance was generally preserved across plausible shifts. Detailed data are presented in eTable 4, Supplement and eTable 5, Supplement.

## Discussion

This RCT found that daily 1 mg/kg iron supplementation between 4 and 9 months of age in exclusively or predominantly breastfed infants may reduce externalizing CBCL scale T-scores and aggressive behaviors at 3 years of age. These findings suggest that low-dose iron supplementation may contribute to reducing behavior problems in young children in settings with low prevalence of ID and IDA.

Observational research strongly suggests that early ID has a negative impact on later behavior^34^. Infants with IDA show increased wariness, hesitancy, reduced engagement, and blunted positive affect compared to iron-sufficient peers^35,36^. By age 4–5 years, they tend to display less positive affect, passivity, lower engagement and poorer self-control in delayed-gratification tasks^27,37,38^. Long-term follow-up studies link early-life ID to increased detachment, poorer emotional health, sluggish cognitive tempo and ADHD symptoms persisting into adolescence and early adulthood^6,39^.

The impact of iron supplementation on behavioral problems remains understudied in interventional research. A systematic review by Pasricha et al. on the effects of daily iron supplementation on health in children aged 4 to 23 months identified 35 eligible RCTs conducted in settings of high risk of anemia, none of which reported behavior as an outcome^40^. Evidence from settings of low anemia prevalence is even more scarce. We have previously reported results from an RCT in low-birth-weight (LBW) infants (*n* = 285), showing that those who received low-dose iron supplementation from 6 weeks to 6 months had significantly lower risk of behavioral problems at age 3 compared to those given placebo with some benefits persisting at age seven^41,42^. Additionally, iron supplementation reduced the risk of early ID and IDA in the LBW infants^43^. Although no reduction in the risk of early ID/IDA was found in this trial of term healthy infants^28^, the observed effect on behavioral problems suggests that supplementation may have improved brain iron availability in ways not detectable by blood biomarkers.

To our knowledge, this study is the first placebo-controlled RCT reporting effects of iron supplementation on behavioral outcome in healthy infants. The shared study settings and comparable improvements observed in our study and the aforementioned RCT by Berglund et al.^41^ in a population at risk of ID strengthen the validity of our findings and suggests that brain iron availability during the early postnatal period may be involved in the processes underlying neurodevelopmental disorders.

The neurobiological mechanisms linking early ID to adverse neurodevelopmental behavioral outcomes remain unclear^44^. Iron is important for regulating neurotransmitter metabolism, particularly dopamine^45–48^. Animal studies indicate that early ID primarily affects the hippocampus, essential for recognition and memory, and causes irreversible disruptions to dopaminergic pathways in the striatum, a region involved in regulating executive functions^49–54^. Similar impairments have been found in humans, where Lukowski et al. reported that young adults with severe ID during infancy had poorer performance in executive functions and a recognition memory task^55^. In this trial, iron supplementation resulted in less aggressive behavior and attention problems, which may suggest dopaminergic pathway being involved.

Neurodevelopmental disorders such as ADHD (externalizing behaviors, attention deficits, hyperactivity, impaired executive functioning) and ASD (internalizing, withdrawn behaviors)^56^ are common and place a substantial burden on society and healthcare^15,57,58^. Both ADHD and ASD are associated with altered dopaminergic pathways^59^. Our findings support evidence that improved early iron availability may influence neurodevelopment^19,60^, potentially benefiting both individuals and society.

The strengths of this study are its well-structured design and methodological transparency. Despite challenges posed by the COVID-19 pandemic, we managed to follow up with the majority of participants. However, stricter regulations in Poland compared to Sweden increased the dropout rate among Polish participants (eTable 6, Supplement) with a total attrition reaching 40%. Participant dropout is a common challenge in long-term nutrition trials^61^. While dropout rates were similar across intervention groups, participants who completed the CBCL had higher birth weights and gestational ages than those lost to follow-up. We addressed the non at random missing data scenario by performing sensitivity analyses which indicated that the primary findings are robust to a wide range of plausible missing data scenarios, with only highly unrealistic assumptions leading to change in primary conclusions.

Although similar dropout across intervention groups suggests minimal impact on comparability, the absence of children with ID/IDA who may exhibit more behavioral issues may have reduced variability and exaggerated the observed effects. Given the available sample size (primarily calculated for the main outcome at 12 months), the study had moderate power to detect differences in CBCL outcomes, with the minimum detectable effect size estimated at 0.49. Therefore, smaller differences may have gone undetected. Despite covariate-adjusted analysis, unmeasured social and environmental factors may also have influenced the outcome measures. Additionally, the use of country-specific CBCL versions and potential differences between countries may have reduced precision due to the nested data structure. Although randomization balanced groups within countries, clustering may have underestimated variability. These factors warrant cautious interpretation and may limit the generalizability of the results.

The findings of this study suggest that early iron supplementation may be a promising nutritional strategy to mitigate behavioral problems at preschool age. This study was motivated by conflicting guidelines on iron supplementation in breastfed infants^13,14^. While we have shown that low-dose daily iron supplementation did not improve psychomotor development^28^, it appears to be associated with fewer behavioral problems. These effects in healthy infants align with findings from high-risk groups for ID. Although the results are promising and may support the recommendation for universal iron supplementation, the evidence remains too limited to justify changes in early feeding practices for the broader population of breastfed infants. Further well-designed and adequately powered trials controlling for environmental factors related to behavioral patterns are required to confirm the findings and their clinical relevance. If causality can be confirmed, possible mechanisms by which mild iron deficiency influences behavioral development should be explored.

## Conclusion

This pre-specified secondary analysis of a randomized trial suggests that low-dose iron supplementation between 4 and 9 months of age in exclusively or predominantly breastfed infants may reduce externalizing behavior problem scores, particularly aggressive behavior, at 3 years of age. Although these findings are promising, cautious interpretation is warranted until they are confirmed by further studies. Nonetheless, these novel findings provide valuable insight into the potential benefits of routine iron supplementation for healthy, exclusively or predominantly breastfed children in settings of low rates of ID and IDA. Furthermore, our results suggest that future trials on early-life iron supplementation may benefit from focusing on neuropsychiatric outcomes rather than traditional neurodevelopmental outcomes.

## Supplementary Information

Below is the link to the electronic supplementary material.

Supplementary Material 1

## Data Availability

Unimputed data will be shared upon reasonable request sent to the principal investigator, following approval of research purposes with appropriate plan of analysis and acknowledgements.
