# Supplementary material for: Iron in breastfed infants and behavior at 3 years: a randomized trial
Source: Sci Rep. 2026 Jun 16;16:18664. doi: 10.1038/s41598-026-56401-z (PMC13272676; doi:10.1038/s41598-026-56401-z)
Supplement: Supplementary file 1 — Supplementary Material 1 [file 41598_2026_56401_MOESM1_ESM.docx]

**eTable 1, Supplement. Internal consistencies^1^ of CBCL broadband and subscale scores, intention-to-treat analysis.**

| **CBCL Scale** | **Classical Cronbach’s α** | | **Polychoric Cronbach’s α** | |
| --- | --- | --- | --- | --- |
|  | **Poland  (n = 38)** | **Sweden  (n = 95)** | **Poland  (n = 38)** | **Sweden  (n = 95)** |
| Broadband scores |  |  |  |  |
| Total T-score | 0.90 | 0.90 |  |  |
| Internalizing T-score | 0.68 | 0.77 |  |  |
| Externalizing T-score | 0.88 | 0.84 |  |  |
| Subscale scores |  |  |  |  |
| I Emotionally Reactive | 0.50 | 0.58 | 0.79 | 0.74 |
| II Anxious/Depressed | 0.49 | 0.55 | 0.69 | 0.83 |
| III Somatic Complaints | 0.39 | 0.34 | 0.79 | 0.83 |
| IV Withdrawn | 0.18 | 0.62 | 0.73 | 0.89 |
| V Sleep Problems | 0.42 | 0.66 | 0.50 | 0.77 |
| VI Attention Problems | 0.58 | 0.53 | 0.56 | 0.72 |
| VII Aggressive Behavior | 0.89 | 0.82 | 0.90 | 0.89 |
| Classical and polychoric Cronbach's alpha are reported. Classical alpha is limited under conditions of minimal variability. Ordinal alpha is a more suitable alternative for CBCL’s response format (ordinal, Likert scale).  ^1^ Separated by the different translated versions of the CBCL (Swedish and Polish) used. | | | | |

**eTable 2, Supplement. CBCL broadband scores, cutoffs**^a^ **and subscale**^b^ **scores between intervention groups, per-protocol analysis.**

| CBCL Scale^c^ | Iron (n = 57) | Placebo (n = 52) | MD or RR (95% CI)^d^ | *p*^e^ | *p*^f^ |
| --- | --- | --- | --- | --- | --- |
| Broadband scores |  |  |  |  |  |
| Total T-score | 44.7 (8.4) | 46.9 (8.6) | -2.17 (-5.37 to 1.04) | **.002**^1^ | **.02** |
| Total T-score above clinical cutoff | 0 (0) | 2 (3.4) | NA | .26^4^ | .49 |
| Total T-score above subclinical cutoff | 2 (3.8) | 4 (8.4) | 0.45 (0.08 to 2.51) | .42^4^ | .68 |
| Total T-score above Swedish cutoff | 4 (7.3) | 8 (14.6) | 0.50 (0.16 to 1.62) | .23^3^ | .49 |
| Internalizing T-score | 43 (33 to 51) | 45 (41 to 51) | -2.17 (-5.73 to 1.39) | .20^2^ | .49 |
| Internalizing T-score above clinical cutoff | 0 (0) | 0 (0) | NA | >.99^4^ | 1 |
| Internalizing T-score above subclinical cutoff | 4 (7.2) | 4 (8.4) | 0.85 (0.21 to 3.42) | >.99^4^ | 1 |
| Internalizing T-score above Swedish cutoff | 5 (9.6) | 6 (11.5) | 0.84 (0.23 to 3.02) | .43^3^ | .68 |
| Externalizing T-score | 45.4 (8.9) | 48.2 (9.5) | -2.85 (-6.31 to 0.61) | **.001**^1^ | **.02** |
| Externalizing T-score above clinical cutoff | 2 (3.5) | 5 (9.6) | 0.36 (0.07 to 1.80) | .26^4^ | .49 |
| Externalizing T-score above subclinical cutoff | 4 (7.7) | 5 (9.6) | 0.80 (0.22 to 2.85) | .73^4^ | .92 |
| Externalizing T-score above Swedish cutoff | 4 (7.7) | 5 (9.6) | 0.80 (0.22 to 2.85) | .73^4^ | .92 |
| Subscale scores |  |  |  |  |  |
| I Emotionally Reactive | -0.26 (-0.89 to 0.99) | 0.36 (-0.26 to 0.99) | -0.19 (-0.69 to 0.30) | .2^2^ | .49 |
| II Anxious/Depressed | 0.08 (-0.64 to 0.81) | 0.08 (-0.64 to 0.99) | 0.00 (-0.46 to 0.46) | .78^2^ | .92 |
| III Somatic Complaints | -0.36 (-0.90 to 0.19) | -0.36 (-0.90 to 0.19) | -0.06 (-0.38 to 0.25) | .83^2^ | .92 |
| IV Withdrawn | -0.62 (-0.62 to 0.10) | 0.10 (-0.62 to 0.10) | -0.22 (-0.52 to 0.07) | .07^2^ | .26 |
| V Sleep Problems | -0.16 (-0.62 to 0.29) | -0.16 (-0.62 to 0.75) | -0.11 (-0.49 to 0.26) | .56^2^ | .82 |
| VI Attention Problems | -0.32 (-0.98 to 0.99) | 0.33 (-0.32 to 0.99) | -0.35 (-0.74 to 0.05) | .06^2^ | .26 |
| VII Aggressive Behavior | -0.05 (1.0) | 0.25 (1.1) | -0.31 (-0.70 to 0.09) | **.004**^1^ | **.03** |
| Abbreviations: MD – Mean difference (iron vs placebo groups); RR – relative risk (iron vs placebo groups); CI – confidence interval; NA – not applicable.  ^a^ Broadband scale cutoffs: clinical (T-Score >63), subclinical (T-Score >59); Swedish cutoff: >90th percentile of a Swedish reference population.^30^  ^b^ Subscale scores standardized to a Swedish reference population.^30^  ^c^ Numeric variables are presented as mean (SD) or median (interquartile range), depending on normality of distribution; categorical variables are presented as pooled % of group based on pooled % and total number of patients in the iron/placebo group.  ^d^ MD for numeric values, RR of scoring above cutoff for categorical variables. Note: Discrepancies between P values and CIs, with some CIs crossing zero despite statistical significance, may occur due to multiple imputation.  ^e^ Groups were compared using (1) Student’s t-test, (2) Mann-Whitney U test, (3) Pearson’s chi-square test, (4) Fisher’s exact test, as appropriate.  ^f^ P values adjusted using the Benjamini-Hochberg method. | | | | |  |

**eTable 3, Supplement. Raw CBCL subscale**^a^ **scores between intervention groups, intention-to-treat analysis.**

| CBCL subscale scores^b^ | Iron (n = 67) | Placebo (n = 66) | *p*^c^ | *p*^d^ |
| --- | --- | --- | --- | --- |
| I Emotionally Reactive | -0.26 (-0.89 to 0.99) | 0.36 (-0.26 to 0.99) | .06^2^ | .18 |
| II Anxious/Depressed | 0.08 (-0.64 to 0.81) | 0.08 (-0.64 to 1.54) | .40^2^ | .58 |
| III Somatic Complaints | -0.36 (-0.90 to 0.19) | -0.36 (-0.90 to 0.19) | .83^2^ | .83 |
| IV Withdrawn | -0.62 (-0.62 to 0.10) | 0.10 (-0.62 to 0.10) | **.02^2^** | .08 |
| V Sleep Problems | -0.16 (-0.62 to 0.29) | -0.16 (-0.62 to 1.20) | .36^2^ | .57 |
| VI Attention Problems | -0.32 (-0.98 to 0.66) | 0.33 (-0.32 to 0.99) | **.02^2^** | .09 |
| VII Aggressive Behavior | -0.02 (0.96) | 0.29 (1.03) | **.003^1^** | **.03** |
| ^a^ Subscale scores standardized to a Swedish reference population.^30^  ^b^ Numeric variables are presented as mean (SD) or median (interquartile range), depending on normality of distribution.  ^c^ Groups were compared using (1) Student’s t-test, (2) Mann-Whitney U test, as appropriate.  ^d^ P values adjusted using the Benjamini-Hochberg method. | | | |  |

**eTable 4, Supplement. Summary of tipping-point analysis^a^ assuming varying value of drop-out patients from iron group and stable value of drop-out patients in placebo group.**

| **Variable** | **Tipping-point (SD shift from mean)** | **Outcome** |
| --- | --- | --- |
| Total T-score & Externalizing T-score | ≤ 0.3 SD | Significant difference (Iron < Placebo) |
|  | 0.4 SD to 1.5 SD | Loss of significance (Iron ~ Placebo) |
|  | ≥ 1.6 SD | Significant difference (Iron > Placebo) |
| Internalizing T-score | ≤ 0.2 SD | Significant difference (Iron < Placebo) |
|  | 0.3 SD to 1.4 SD | Insignificant difference (Iron ~ Placebo) |
|  | ≥ 1.5 SD | Significant difference (Iron > Placebo) |
| ^a^ Iron drop-outs: mean + SD shift; Placebo drop-outs: mean. | | |

**eTable 5, Supplement. Summary of tipping-point analysis^a^ assuming varying value of drop-out patients from placebo group and stable value of drop-out patients in iron group.**

| **Variable** | **Tipping-point (SD shift from mean)** | **Outcome** |
| --- | --- | --- |
| Total T-score & Externalizing T-score | ≤ -1.5 SD | Significant difference (Iron > Placebo) |
|  | -1.4 SD to -0.4 SD | Loss of significance (Iron ~ Placebo) |
|  | ≥ -0.3 SD | Significant difference (Iron < Placebo) |
| Internalizing T-score | ≤ -1.6 SD | Significant difference (Iron > Placebo) |
|  | -1.5 SD to -0.3 SD | Loss of significance (Iron ~ Placebo) |
|  | ≥ -0.2 SD | Significant difference (Iron < Placebo) |
| ^a^ Iron drop-outs: mean; Placebo drop-outs: mean + SD shift. | | |

**eTable 6, Supplement. Sociodemographic, perinatal and background information, per country of origin.**

| **Characteristic, n (%)** | **Whole population**^a^ | | **With available**^b^ **CBCL data**^c^ | |
| --- | --- | --- | --- | --- |
|  | **Polish (n = 112)** | **Swedish (n = 109)** | **Polish (n = 38)** | **Swedish (n = 95)** |
| Intervention group |  |  |  |  |
| Placebo | 56 (50) | 54 (49.5) | 19 (50) | 47 (49.5) |
| Iron | 56 (50) | 55 (50.5) | 19 (50) | 48 (50.5) |
| Sex |  |  |  |  |
| Male | 60 (53.6) | 50 (45.9) | 16 (42.1) | 44 (46.3) |
| Female | 52 (46.4) | 59 (54.1) | 22 (57.9) | 51 (53.7) |
| Infant characteristics, mean (SD) |  |  |  |  |
| Gestational age | 39.2 (1.3) | 39.7 (1.3) | 39.5 (1.3) | 39.8 (1.2) |
| Birth weight | 3471.6 (361.1) | 3631.7 (418.9) | 3539.1 (364.9) | 3639 (405) |
| Birth head circumference | 34.9 (1.5) | 35 (1.3) | 35 (1.6) | 35 (1.2) |
| Maternal education level |  |  |  |  |
| Compulsory school | 2 (2.3) | 20 (18.5) | 0 (0) | 18 (19.1) |
| Upper secondary or college | 0 (0) | 14 (13) | 0 (0) | 12 (12.8) |
| University or higher | 86 (97.7) | 74 (68.5) | 35 (100) | 64 (68.1) |
| Paternal education level |  |  |  |  |
| Compulsory school | 3 (3.4) | 2 (1.9) | 1 (2.9) | 2 (2.2) |
| Upper secondary or college | 14 (15.9) | 42 (39.3) | 6 (17.1) | 37 (39.8) |
| University or higher | 71 (80.7) | 63 (58.9) | 28 (80) | 54 (58.1) |
| Breastfeeding |  |  |  |  |
| Breastfeeding at 6 months | 41 (50) | 97 (92.4) | 17 (53.1) | 88 (92.6) |
| Exclusively breastfed at 6 months | 37 (45.1) | 66 (62.9) | 14 (43.8) | 59 (62.1) |
| Breastfeeding at 9 months | 10 (17.2) | 70 (69.3) | 5 (21.7) | 62 (67.4) |
| >50% breastfed at 9 months | 6 (60) | 17 (24.3) | 3 (60) | 16 (25.8) |
| Iron deficiency at 12 months | 12 (16.7) | 2 (2.9) | 1 (3.7) | 2 (3.1) |
| Iron deficiency anemia at 12 months | 2 (3.3) | 0 (0) | 0 (0) | 0 (0) |
| ^a^ Available data on each variable in Polish/Swedish participants: 112/109 (Intervention group, sex), 110/109 (birth weight), 108/109 (gestational age), 101/95 (birth head circumference), 88/108 (maternal education) 88/107 (paternal education), 82/105 (breastfeeding at 6 months), 81/82 (ferritin), 77/78 (C-reactive protein), 75/75 (hemoglobin), 72/69 (ID), 61/60 (IDA), 58/101 (breastfeeding at 9 months).  ^b^ Complete CBCL was available for 104 participants (35/69); multiple imputation was used for those with incomplete CBCL data (3/26).  ^c^ Available data on each variable in Polish/Swedish participants: 38/95 (Intervention group, sex, gestational age, birth weight), 34/85 (birth head circumference), 35/94 (maternal education), 35/93 (paternal education), 32/95 (breastfeeding at 6 months), 30/74 (ferritin), 29/71 (C-reactive protein), 29/67 (hemoglobin), 27/64 (ID), 27/65 (IDA), 23/92 (breastfeeding at 9 months). | | | | |
